# Use of the Signature Fatty Acid 16:1**ω**5 as a Tool to Determine the Distribution of Arbuscular Mycorrhizal Fungi in Soil

**DOI:** 10.1155/2012/236807

**Published:** 2012-07-04

**Authors:** Christopher Ngosong, Elke Gabriel, Liliane Ruess

**Affiliations:** ^1^Institute of Biology, Ecology Group, Humboldt-Universität zu Berlin, Philippstraße 13, 10115 Berlin, Germany; ^2^Department of Natural Resource Sciences, MacCampus, McGill University, 21,111 Lakeshore Road, Ste Anne de Bellevue, QC, Canada H9X 3V9; ^3^Institute of Vegetable and Ornamental Crops Großbeeren, Theodor-Echtermeyer-Weg 1, 14979 Großbeeren, Germany; ^4^Faculty of Food and Agriculture, UAE University, Jimi 1 Campus, Building 52, P.O. Box 17555, Al Ain, Abu Dhabi, UAE

## Abstract

Biomass estimation of arbuscular mycorrhiza (AM) fungi, widespread plant root symbionts, commonly employs lipid biomarkers, predominantly the fatty acid 16:1**ω**5. We briefly reviewed the application of this signature fatty acid, followed by a case study comparing biochemical markers with microscopic techniques in an arable soil following a change to AM non-host plants after 27 years of continuous host crops, that is, two successive cropping seasons with wheat followed by amaranth. After switching to the non-host amaranth, spore biomass estimated by the neutral lipid fatty acid (NLFA) 16:1**ω**5 decreased to almost nil, whereas microscopic spore counts decreased by about 50% only. In contrast, AM hyphal biomass assessed by the phospholipid (PLFA) 16:1**ω**5 was greater under amaranth than wheat. The application of PLFA 16:1**ω**5 as biomarker was hampered by background level derived from bacteria, and further enhanced by its incorporation from degrading spores used as microbial resource. Meanwhile, biochemical and morphological assessments showed negative correlation for spores and none for hyphal biomass. In conclusion, the NLFA 16:1**ω**5 appears to be a feasible indicator for AM fungi of the Glomales group in the complex field soils, whereas the use of PLFA 16:1**ω**5 for hyphae is unsuitable and should be restricted to controlled laboratory studies.

## 1. Introduction

The chemotaxonomic use of lipids has a long tradition in microbiological research [1–3]. Due to the different enzymatic capabilities in lipid metabolism, fatty acids display a great structural diversity and biological specificity, providing an integrated and quantitative measure of microbial biomass and community structure in different environments [4]. In particularly, PLFAs have been employed in soil ecosystems as biomarkers for bacteria, saprotrophic fungi, and AM fungi; see Zelles [5] and Joergensen and Wichern [6] for detailed reviews. Moreover, as the lipid pattern of faunal consumers reflects the fatty acid composition of their diet, trophic biomarker fatty acids for major food resources in soil decomposers have been assigned [7].

Bacteria and fungi are important drivers of soil processes, predominantly nutrient mineralization and transfer to plants. Among the different mycorrhizal types, arbuscular fungi that form symbiosis with the roots of about 80% of all vascular plants are the dominant fungal symbionts that support plant growth [8, 9]. The AM fungal extraradical mycelium (ERM) spreads beyond the rhizosphere of host plants, providing additional surface area for the acquisition of phosphorus and nitrogen [10–12]. In recent years, global interest in sustainable agricultural practices has led to increase in the production and application of AM fungal inoculum in arable soils, which requires reliable methods for their quantification. AM fungi may occur naturally in arable soils, but their density and diversity may be increased by farm management practices such as fertilization or crop types [13–15].

Despite the importance of AM fungi for soil nutrient cycling, information on their distribution is inadequate due to analytical difficulties that are limiting the ability to study processes at the microscale. Four main approaches for quantifying fungal contribution to soil microbial communities are commonly used in soil ecology: (1) microscopic methods, (2) selective inhibition, (3) specific cell membrane components, and (4) specific cell wall components, with microscopic methods generally recording the lowest values [6]. Classical morphological estimation of AM fungal communities in soil includes membrane filter [16], grid line intersection [17], spore counts [3], and aqueous filtration extraction method and quantification of extraradical mycelium based on morphological criteria [18]. Molecular techniques have also been developed to determine AM fungi [19, 20], but difficulties involved in the sequencing as well as the absence of a sequence for some species constrain the use of this approach. More advanced, and particularly for quantification of AM fungal biomass, is the lipid biomarker technique that is being applied to determine the distribution of AM fungi in soil over the last two decades.

Olsson et al. [21, 22] were the first to use signature fatty acid 16:1*ω*5 regularly to assess growth and interactions of AM fungi in experimental soil-plant systems (Table 1). They proposed the 16:1*ω*5 as marker fatty acid, with the PLFA fraction assigning viable fungal hyphal biomass, and the NLFA fraction determining storage lipids such as spores [23]. This biomarker approach was also adopted for mycelial structures within plant roots, that is, the NLFA for energy storage in vesicles and the PLFA for membrane constituents such as intraradical mycelium or arbuscles [24, 25]. Based on this, both the 16:1*ω*5 PLFA and NLFA are widely used as indicators for AM fungi across soil ecosystems (Table 1). In a recent review, Joergensen and Wichern [6] proposed 345 as conversion factor of nmol PLFA to biomass C of AM fungi. This factor, a weighted mean based on literature data originating from four studies, can be criticized. However, it demonstrates the common use of 16:1*ω*5 PLFA in AM investigations, even though its biomarker value may be hampered.

Despite the proposed primary origin of 16:1*ω*5 PLFA, it may additionally be found, although in smaller amounts, in the lipids of other organisms, particularly in soil-inhabiting bacteria [39, 40]. On the other hand, the 16:1*ω*5 NLFA is not only present in spores but also forms the transport vehicle for carbon between intraradical and extraradical mycelium [41]. As assigned by ^13^C labelling studies, the fungus converts sugars taken up in the root compartment into lipids [42, 43], which can be used to assess the shift of carbon from roots into associated microbial communities without extraction, purification and identification of fungal mycelium [44, 45]. These stable isotope studies indicated that the NLFA 16:1*ω*5 is a good tool to assign AM fungal biomass in soil but showed no evidence for the application of the PLFA fraction. Despite these discrepancies several recent studies employed the PLFA marker for AM extraradical mycelium in field soils (Table 1).

This general application of 16:1*ω*5 as biomarker for AM fungi across different soil ecosystems calls for deeper insight to ensure reliability in the quantification of the fungal hyphae or spore biomass. We therefore performed a case study on the dynamics of AM fungal spores and extraradical mycelium comparing lipid biomarker and morphological approaches to determine the distribution of AM fungi in an arable field soil. A long-term fertilizer experiment was used, with the shift to a nonmycorrhizal host plant after 27 years of host crop cultivation. This unique experimental design offers the possibility to assess whether biochemical and microscopic techniques yield similar results under distinct changes in the mycorrhizal symbiosis *in situ*, without artificial manipulations (e.g., selective inhibition technique). Investigating the same plots in two successive vegetative periods allowed (i) to assign the diminishing of AM fungi in the absence of a host plant, (ii) to screen the background signal from the remaining vegetation period using both methods, and (iii) to determine correlations between the changes within biochemical marker and morphological assessments.

## 2. Materials and Methods

### 2.1. Field Site

The study was conducted at a long-term arable field site established in 1980 at the Institute of Biodynamic Research (IBDF) Darmstadt in Germany. The field site is located at 49°N, 8°E, and 100 m above sea level, with annual mean air temperature of 9.5°C and precipitation of 590 mm. The soil type is haplic cambisol comprising 87% sand, 8% silt, and 5% clay in the topsoil. The experimental setup was a two-factorial design amended with mineral (NPK) and organic (cattle manure + biodynamic preparations) fertilizers applied at low and high amounts. These were implemented in a split block design with four replicate plots of 5 m × 5 m each. Except fertilization, all other farming practices such as irrigation, tillage, and crop rotation were similar across the 27 years since the establishment of the long-term field experiment. Plots amended with mineral fertilizer received N, P, K applied in rates of kg ha^−1^y^−1^ as 60, 50, 75 (low), and 140, 100, and 125 (high), respectively. Organic plots received composted cattle manure with the addition of biodynamic preparations spread as solid fertilizer before ploughing and milling of the soil. The application rate was calculated to achieve similar nitrogen input as at the mineral fertilizer plots, which resulted in the variation of phosphorus and potassium amounts depending on the manure properties of a given year. On average, organic plots received less P (−25% for low and −38% for high) and 26% more K than mineral plots. For more details on farm management, see Ngosong et al. [46].

### 2.2. Sampling

Since the long-term field site was established, there has been 27 years continuous mycorrhizal host crop rotation including lupine angustifolius, winter rye, potato, spring wheat, and clover. The present investigations were conducted during two successive cropping seasons with the cultivation of spring wheat (*Triticum aestivum* cv. Passat) in 2007 and a shift to amaranth (*Amaranthus hypochondriacus*) in 2008. The former is a well-known host plant for AM fungi while the latter is recognized as non-host [8, 47]. Soil at the wheat plots was sampled four months after sowing and three weeks before crop harvest, while amaranth plots were sampled two months after sowing and two months before crop harvest. For examination of AM fungal morphological structures (hyphal length, spores) and lipids (PLFAs, NLFAs), one soil sample was taken from each replicate plot (*n* = 4 per treatment) at 0–5 and 5–10 cm depth using 5 cm diameter soil core. Soil was stored at −20°C prior to analyses. Additionally, random samples of wheat and amaranth roots were collected from the respective plots and analysed for infection by AM fungi.

### 2.3. Morphological AM Fungal Investigation

Morphological assessment of AM fungal structures comprised the colonization of crop roots, length of extraradical mycelium, and number of spores in the bulk soil. For the assessment of root infection rate, fine roots (0.7–1.0 g) were stained with trypan blue in lactic acid and the colonized roots assessed by modified intersection method with 250–300 intersections counted per sample [17]. Fungal spores were isolated from 80 g air-dried soil by sieving and decanting method, with subsequent sucrose gradient centrifugation [48], and counted using the agar film technique [49].

The hyphal length was estimated from soil using a modified membrane filtration technique. Soil sample (1.0 g) was homogenized with 100 mL deionized water in a laboratory mixer (Waring Commercial; Connecticut, USA) for 60 seconds. The suspension was poured through a 40-micrometer filter and washed carefully with water to eliminate fine soil particles. Remaining material was transferred into a petri dish and stained with a few drops of 0.05% trypan blue in lactic acid. The suspension was transferred into a glass beaker and diluted to 300 mL volume. A subsample of the suspension was filtered on a 0.45 *μ*m mesh width membrane filter (MicronSep; GE Water & Process Technologies, USA) using a bottleneck filtration unit (NALGENE Reusable Bottle Top Filter Unit; Nalge Company, New York, USA). The membrane filter was mounted onto microscopic slides and observed under the microscope at 200x magnification, and the AM fungal hyphal length estimated by a modified gridline intersection method [50, 51].

### 2.4. Fatty Acid Analysis

Lipids were extracted from 4 g soil (wet weight) using the modified Bligh and Dyer method according to Frostegård et al. [52]. Fractionation into NLFAs, glycolipids, and PLFAs was performed using silica acid columns (HF BOND ELUT–SI, Varian Inc.), and the different fractions were eluded with chloroform, acetone, and methanol, respectively. Lipid methanolysis of PLFA and NLFA fractions was conducted in 0.2 M methanolic KOH, and methyl nonadecanoate (19 : 0) was added as internal standard; for more details see Ngosong et al. [53].

Fatty acid methyl esters (FAMEs) were identified by chromatographic retention time comparison with a standard mixture composed of 37 different FAMEs ranging from C11 to C24 (Sigma-Aldrich, St Louis, MO, USA). Analysis was performed by gas chromatography using a GC-FID Clarus 500 (PerkinElmer Corporation, Norwalk, USA) equipped with HP-5 capillary column (30 m × 0.32 mm i.d., film thickness 0.25 *μ*m). To verify correct identification of FAMEs (chain length and saturation), a range of soil samples were analyzed by mass spectrometry using a 3400/Saturn4 Diontrap GC/MS system (Varian, Darmstadt, Germany), equipped with a HP-5 capillary column (50 m × 0.32 mm i.d., film thickness 0.17 *μ*m). A mass range from 50 to 500 m/z was monitored twice a second in Scan mode; for more details see Ngosong et al. [46]. The signature fatty acid 16:1*ω*5 was used as biomarker for AM fungi, where the PLFA fraction represents fungal extraradical mycelium and the NLFA spore for biomass [23, 54, 55].

### 2.5. Statistical Analysis

The effects of crop plant shift on AM fungal fatty acid marker and morphological estimations were tested using STATISTICA 6.0 for Windows [56]. Data were subjected to nonparametric statistics using Kruskal-Wallis. Significant effects (*P* < 0.05) of the different factors are indicated in figures. Additionally, the Spearman Rank Order Correlations between biochemical and microscopically derived results were performed.

## 3. Results

**Figure 1:**
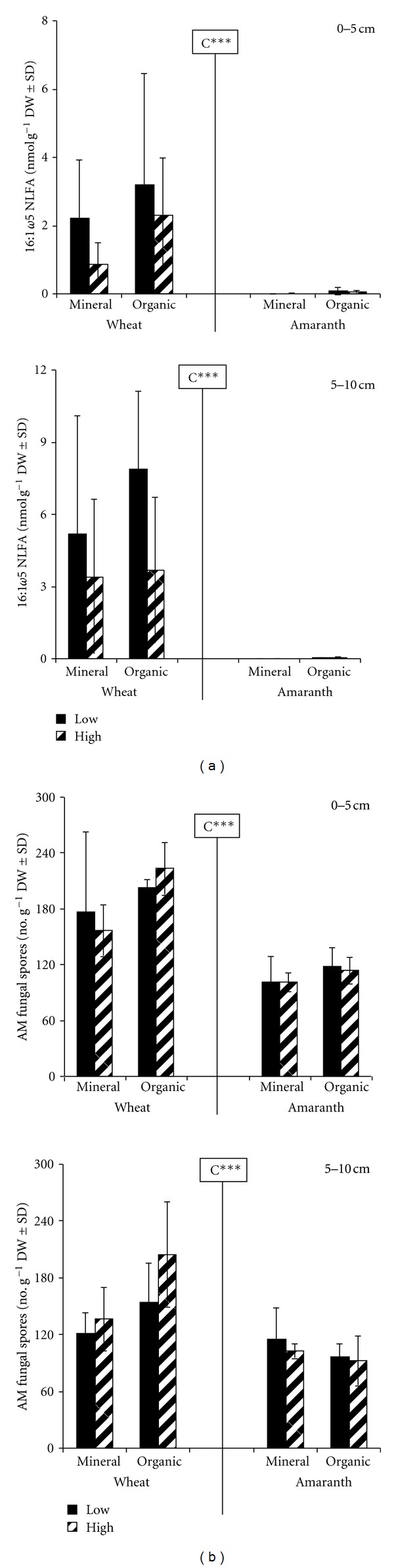
Comparison of microscopic and biochemical estimations of arbuscular mycorrhiza spores in an arable field soil cultivated with wheat or amaranth as crop; 0–5 and 5–10 cm soil depths; amendments with mineral (NPK) and organic (cattle manure + biodynamic preparations) fertilizers applied at low and high amounts; C = crop type; Kruskal-Wallis with *** for *P* < 0.001. (a) AM spores assessed by neutral lipid fatty acid 16:1*ω*5 (nmol g^−1^ DW soil ± SD), (b) Spore density counts (No. g^−1^ DW soil ± SD).

The morphological examination of crop roots revealed 32–67% colonization of wheat by AM fungi, whereas the nonmycorrhizal host amaranth was not infected (data not presented). This clearly indicates the absence of an active amaranth-fungal symbiosis. In conformity, in the absence of a host plant, AM fungal spore biomass assessed by the NLFA 16:1*ω*5 almost disappeared in amaranth soils, with less than 0.1 nmol g^−1^ DW, compared to 0.9–7.9 nmol g^−1^ DW for wheat soils across depths (Figure 1(a)). Similarly, the microscopic counted spore numbers decreased, but only by 55%, and ranged between 121 and 205 and 87 and 125 spores g^−1^ DW soil for wheat and amaranth plots, respectively (Figure 1(b)). The relationship between NLFA and microscopic spore estimates was negatively correlated (*r* = −70, *P* < 0.05) across fertilizers, depths, and crop plant. Overall, the estimation of AM fungi in the arable soil using signature fatty acid and microscopic techniques mirrored the same trend, but to a different extent.

In contrast to AM fungal spores, hyphal biomass assessed by the marker PLFA 16:1*ω*5 increased significantly under amaranth with 0.4–1.1 nmol g^−1^ DW compared to 0.1–0.8 nmol g^−1^ DW for wheat soil; see Ngosong et al. [46]. This corresponds to an increase by 24–65% in the upper soil and 39–79% in the lower soil layer at amaranth plots. Meanwhile, AM hyphal length under amaranth determined morphologically ranged between 1.5 and 4.0 m g^−1^ DW and 1.8 and 2.5 m g^−1^ DW soil at 0–5 and 5–10 cm depth, respectively (Table 2). This contradicts the absence of amaranth root infection by the fungus, and the strong decrease in spore numbers at those plots. Nonetheless, there was no observed correlation (*r* = −0.13, *P* > 0.05) between morphological AM hyphal length and biochemical PLFA 16:1*ω*5 hyphal biomass under amaranth across fertilizer types and soil depth. In addition, there was no correlation (*r* = −0.10, *P* > 0.05) between wheat root infection rate and the PLFA 16:1*ω*5 at wheat plots.

## 4. Discussion

The present investigation focuses on the correlation of morphological and biochemical estimates of AM fungal dynamics in light of the shift from host to non-host crops. The response of microbial communities, including AM fungi, in relation to fertilizer type and amount as demonstrated by lipid data is discussed in detail elsewhere [46]. When comparing the NLFA signature fatty acid with microscopic estimations, both approaches mirrored the same trend but to a different extent. For the fatty acid, the decline in spore biomass without a host plant was severe with almost nil left, whereas the number of spores remaining was about 50%. Firstly, these differences may be due to low NLFA yield since Olsson [23] suggested that, for efficient extraction of lipids, the spore wall must be broken. On the other hand, Madan et al. [57] reported only small and nonsignificant impact when spores were crushed before analysis. Secondly, Olsson and Johansen [58] demonstrated that AM fungal hyphae contain a significant portion of the NLFA 16:1*ω*5 used for carbon transport in lipids. Since hyphae are decomposed much faster than spores, this may have contributed to the diminishing of the NLFA signal within one crop cycle. However, as spores form 90% of the external fungal tissue and 20% of spore mass is NLFA [58], the impact of lipids from extraradical hyphae appears rather low. Thirdly, the signature fatty acid 16:1*ω*5 is common in *Glomales*, whereas it is rare or lacking in other groups such as *Gigaspora* [54, 59]. For the latter, several long chain fatty acids such as 20:1*ω*9 20:2*ω*6 and 22:1*ω*9 have been proposed as biomarkers [57, 60]. Hence, the signature fatty acid partially reflects the dynamics of AM in soil, but not of the entire fungal population. This is supported by the observation of larger spores (e.g., *Gigaspora*) during microscopic examination, which cannot be detected by 16:1*ω*5. Fourthly, microscopic counts are constrained by the fact that newly formed fungal spores are not distinguishable from those formed earlier in the season [61, 62], resulting in potential leftovers from the previous crop. In sum, the NLFA 16:1*ω*5 reflected the decline of AM fungal spores after the change to a non-host crop, but it predominantly represented the *Glomalen* species within the population. Hence, it represents a reliable quantitative estimate of the fungal spore biomass when used in that regard, which is in line with recent studies that applied stable isotopes to assess carbon transfer from roots to AM fungi [44, 45].

In contrast to AM fungal spores, hyphal biomass assessed by the PLFA 16:1*ω*5 biomarker was higher in the non-host compared to the host crop soils. This is surprising as PLFAs are easily decomposed through enzymatic actions in soil and thus are assumed to reflect the occurrence of living organisms [2]. Meanwhile, the longevity of AM hyphae in soil has rarely been measured although it is assumed to be short. Staddon et al. [63] assigned a high turnover rate with an extraradical hyphal live from 5 to 6 days only. On the other hand, Steinberg and Rillig [64] reported that even under relatively favorable conditions for decomposition (18°C; 15% moisture) about 60% of hyphal length were still present 150 days after being separated from their host. However, there was no correlation of the PLFA estimates to hyphal length in amaranth soil, or to root infection rate at wheat plots, indicating a weak relationship between morphological and biochemical measurements. The significant increase of the PLFA 16:1*ω*5 at amaranth compared to wheat plots by up to 79% [46] suggests that bacteria used degrading spores as carbon source, thereby assimilating the marker fatty acid. Such trophic transfer of lipids between microorganisms and their substrates was frequently reported [7]. The extraradical mycelium has been assigned as large and rapid mycorrhizal pathway of carbon into other rhizosphere microorganisms [44, 45, 63]. Our results indicate that also fungal spores are attractive resources that form a considerable microbial carbon pool in the bulk soil.

In conclusion, investigating the development of AM fungi in an arable soil following the change of host crop revealed strong analytical discrepancies between biochemical and microscopic techniques. For the application of PLFA 16:1*ω*5, the background concentration derived from other soil organisms, and particularly bacteria can be too high to correctly quantify mycelium in microbial active soils. Meanwhile, the NLFA 16:1*ω*5 appears to be a reliable marker for AM fungal storage lipids such as spores, yet it cannot assign other than the *Glomales* group. Moreover, the occurrence of NLFA 16:1*ω*5 in extraradical mycelium in soil can superimpose on the overall signal. Clearly, the interpretation that NLFA biomarker arises solely from spores, and PLFA biomarker from mycelia is gross oversimplification. Meanwhile, the approach to combine both the phospholipid and neutral lipid fractions, as marker for AM fungi is no remedy, since it is hampered by the assimilation of signature fatty acid by other decomposers. Overall, these results strongly challenge the use of AM biomarkers, necessitating more comparative *in situ* based studies to identify their structural and functional origin, in order to effectively assign the dynamics of 16:1*ω*5 in complex field soils.

## Figures and Tables

**Table 1:** Application of the marker fatty acid 16:1*ω*5 to assess the distribution of arbuscular mycorrhiza fungi in artificial and natural soil systems. NLFA: neutral lipid fatty acid, PLFA: phospholipid fatty acid.

Authors	Lipid fraction and application (marker for)	Soil system
Olsson et al. [21]	NLFA-storage lipids	Plant mesocosms with *γ* radiated or autoclaved soil
Olsson et al. [22]	PLFA hyphae; conversion factor 38 for nmol PLFA to fungal hyphal length is given	

	NLFA-energy storage (vesicles)	
Larsen and Bødker [24] Van Aarleand Olsson [25]	PLFA-membrane constituents (hyphae, arbuscles)	Mycelia structures in plant roots
	NLFA/PLFA-storage status of fungi	

Olsson and Wilhelmsson [26]		
Hedlund [27]		
Balser et al. [28]		
Hebel et al. [29]	PLFA-hyphal biomass	Grassland, mixed-wood forest stands, arable land, sand dunes; burned forest soil
Huang et al. [30]		
Royer-Tardif et al. [31]		
Marshall et al. [32]		

Olsson and Wilhelmsson [26] Hedlund [27]	NLFA-storage lipids	Arable land, sand dunes
van Groenigen et al. [33] Yao and Wu [34]	NLFA-fungal biomass	Arable land, grassland
Bradley et al. [35]	Total lipids (NLFA + PLFA) fungal biomass	Grassland

Aliasgharzad et al. [36]	NLFA/PLFA separation between arbuscular mycorrhizal fungi (high ratio) and bacteria (low ratio)	Seminatural sandy grassland

Olsson et al. [37] Schnoor et al. [38]	NFLA-^13^C allocation in fungal storage lipids	Pot soil with plants in greenhouse

**Table 2:** Arbuscular mycorrhizal (AM) extraradical mycelium length and hyphal biomass in amaranth plots estimated morphologically and by phospholipid fatty acid biomarker 16:1*ω*5 (% DW soil ± SD), respectively, at 0–5 and 5–10 cm soil depths, amended with mineral (NPK) and organic (cattle manure + biodynamic preparations) fertilizers, applied at low and high amounts.

	Mineral		Organic	
	Low	High	Low	High
0–5 cm				
PLFA 16:1*ω*5 (nmol g^−1^ dry soil)	0.43 ± 0.22	0.55 ± 0.22	0.63 ± 0.07	1.13 ± 0.66
Hyphal length (m g^−1^ dry soil)	3.96 ± 2.39	1.53 ± 0.70	2.08 ± 1.12	2.35 ± 1.36

5–10 cm				
PLFA 16:1*ω*5 (nmol g^−1^ dry soil)	0.51 ± 0.18	0.53 ± 0.3	0.76 ± 0.17	0.71 ± 0.14
Hyphal length (m g^−1^ dry soil)	2.46 ± 1.37	1.78 ± 1.16	1.82 ± 1.01	1.99 ± 1.71
